# Pediatric asthma control during the COVID‐19 pandemic

**DOI:** 10.1002/iid3.418

**Published:** 2021-03-03

**Authors:** Valentina Agnese Ferraro, Andrea Zamunaro, Silvia Spaggiari, Daniela Di Riso, Stefania Zanconato, Silvia Carraro

**Affiliations:** ^1^ Department of Womenʼs and Childrenʼs Health University of Padova Padova Italy; ^2^ Department of Developmental Psychology and Socialization University of Padova Padova Italy

**Keywords:** asthma control, COVID‐19 lockdown, COVID‐19 pandemic, maintenance therapy, pediatric asthma

## Abstract

**Background:**

The lockdown imposed by the COVID‐19 pandemic resulted in a completely different style of life with possible effects on the attitude toward their disease in patients with chronic lung disease, such as asthma. The aim of our study was to investigate in asthmatic children the level of asthma control and the maintenance therapy used during the lockdown.

**Methods:**

Among asthmatic children attending our clinic, we identified those who had been prescribed the same therapy in March‐April 2019 and March‐April 2020. The level of asthma control (GINA‐score) and the maintenance therapy used during the lockdown (March‐April 2020) were compared with those of March‐April 2019. We separately analyzed a small group of children with severe asthma treated with Omalizumab during the lockdown.

**Results:**

We enrolled 92 asthmatic children (67 males). Compared to 2019, in 2020 a higher proportion of children modified their maintenance therapy (38% vs. 15.2%, *p* < .001), with a significant increase in both the proportion of children who increased (*p* = .033) and in that of children who decreased their therapy (*p* = .026). The level of control resulted as significantly higher in 2020 (March *p* = .023; April *p* = .007). Also, the 13 children treated with Omalizumab showed a good level of control in 2020.

**Conclusions:**

In asthmatic children, the COVID‐19 pandemic lockdown had a significant impact on their asthma control and on their attitude toward maintenance therapy.

## Graphical abstract

Our research showed a significant impact of the COVID‐19 pandemic lockdown in the north‐east of Italy on asthmatic children. In particular: (1) The level of asthma control resulted improved during the lockdown period, likely because of the reduced exposure to typical asthma triggers due to the confinement; (2) The maintenance treatment resulted reduced in some patients, likely because of the good level of disease control, and increased in other patients, either because of symptoms or because of fear and anxiety related to the spreading of the COVID‐19 pandemic.

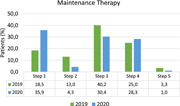

## 1 KEY MESSAGE

In this manuscript, we investigated in asthmatic children the level of asthma control and the use of maintenance therapy during the lockdown imposed by the COVID‐19 pandemic. Our research showed a significant impact of the COVID‐19 pandemic lockdown in the north‐east of Italy on asthmatic children. In particular, the level of asthma control resulted improved during the lockdown period, likely because of the reduced exposure to typical asthma triggers due to the confinement. Also, the maintenance treatment resulted in a reduction in some patients, likely because of the good level of disease control, and increased in other patients, either because of symptoms or because of fear and anxiety related to the spreading of the COVID‐19 pandemic.

The results of our study shed light on the asthma clinical course in children during the COVID‐19 lockdown and on the attitude of children and families toward their asthma treatment during the spreading of COVID‐19, a disease primarily affecting respiratory health.

## 2 INTRODUCTION

The novel coronavirus disease (COVID‐19), induced by SARS‐CoV‐2 (severe acute respiratory syndrome coronavirus 2), has spread since December 2019 from Wuhan, China, and quickly became a pandemic in March 2020.^1^

On the 23rd and 24th of February, the Italian National Health Service reported the first two COVID‐19 cases in two small towns in the Lombardy and Veneto regions. The Italian government promptly reacted by establishing two “red zones” where lockdown with strict restrictions of people movements were imposed and schools, shops, and industrial activities were closed.^2^ With the spread of the pandemic, these extraordinary measures were extended to the whole country on the 11th of March 2020.^2^ Children's lives were profoundly disrupted by the lockdown as the schools were closed and all the extracurricular activities interrupted. These restrictive measures, although necessary, resulted in a completely different style of life, characterized by a high prevalence of psychological distress, manifested most frequently by low mood and irritability, associated with insomnia, posttraumatic stress, and depressive symptoms.^3^ Home confinement disrupted daily routine, increased time of access to the internet and social media, impaired social relations, and reduced outdoor and in‐gym physical activity.^4^

In addition, patients with a chronic lung disease suffered from the fear of being at increased risk of a severe form of COVID‐19 and the fear of having sequelae after a severe COVID‐19 episode.^5^ In fact, in the initial phases of the pandemic, patients with chronic lung diseases, including moderate‐severe asthma and allergy were considered at a potential higher risk of developing severe COVID‐19 than otherwise healthy people.^6^

The restrictions to normal activities, the fear of severe disease in case of Sars‐COV‐2 infection, the reduced accessibility to outpatient clinics due to the change in hospital organization to face COVID‐19 pandemic, represented altogether a completely new scenario likely affecting the perception and attitude toward their disease in asthmatic children and their families.

The main aim of this study was to evaluate during the lockdown imposed by COVID‐19 pandemic the level of asthma control and the adherence to prescribed therapy in asthmatic children, retrospectively analyzing the same period of the previous year for comparison. The secondary aim was the analysis of allergic rhinitis control during the lockdown imposed by COVID‐19 pandemic in a subgroup of asthmatic children affected also by rhinitis. The tertiary aim was to assess psychological functioning during the lockdown in a subgroup of the recruited asthmatic children.

## 3 MATERIALS AND METHODS

We reviewed the clinical records of the asthmatic children, who attended the outpatient clinic of our Unit of Pediatric Allergy and Respiratory Medicine between May 1, 2019 and July 30, 2019. This time frame was chosen to retrieve from the records detailed information on asthma control and use of asthma medication during the months before the visit and, in particular, over March and April 2019. To avoid a possible therapy‐related bias in the comparison of asthma control between 2019 and 2020, we selected children that at the evaluation in 2019 had been judged stable and therefore confirmed on the same maintenance therapy. Children with a personal history of chronic diseases other than asthma were excluded.

In May 2020, the children were reassessed and we collected the information on their asthma control and the use of antiasthma medication during March and April 2020 (months of complete lockdown in Italy). According to Global database on the Implementation of Nutrition Action (GINA) guidelines,^7^ asthma was classified as well‐controlled (GINAscore = 0), partially controlled (GINAscore = 1–2) or uncontrolled (GINAscore = 3–4) depending on the presence of daytime symptoms, night awakening, need for relievers, and limitation to physical activity. In addition, in March and April 2020, the Asthma Control Test (ACT)^8^, ^9^ was administered, evaluating activity limitation, shortness of breath, night‐time symptoms, use of rescue limitation, and patient overall rating of asthma control over the previous four weeks. Higher scores indicate better asthma control.

Furthermore, the occurrence of asthma exacerbations (AEs), defined as a worsening in asthma symptoms requiring a course of at least 3 days of oral steroids, was investigated. The antiasthma therapy taken was classified according to the treatment steps reported in GINA guidelines.

We also asked whether the included children had contracted COVID‐19 or if they had undergone nasal swab for SARS‐CoV‐2 up to the moment of our evaluation.

In children more than 12 years old, suffering from allergic rhinitis, also information on the level of control of this condition was collected for both the analyzed periods. Rhinitis symptom control was assessed using the Rhinitis Control Assessment Test (RCAT),^8^, ^10^, ^11^ with higher scores indicating better rhinitis symptom control. The following items were analyzed: frequency of nasal congestion, sneezing, and watery eyes; sleep disruption; activity limitation caused by symptoms; and self‐rating of symptom control.

In a subgroup of patients, psychological symptoms were assessed through the completion of the Strengths and Difficulties Questionnaire—children's version (SDQ),^12^ a self‐report questionnaire with a 3‐point Likert scale (0 = not at all, 1 = a little, 2 = very much) that asks about 25 attributes to analyze the Total Difficulties Score (TDS). The Questionnaire is made up of the following five subscales: Emotional Symptoms (EMO), Conduct Problems (COND), Hyperactivity–Inattention (HYPER), Peer Problems (PEER), Prosocial Behavior (PROS). Due to the unexpected nature of the COVID‐19 pandemic, the questionnaire was not administered in 2019. Nonetheless, the questionnaire administered in 2020 asked the patients to compare their psychological well‐being with the period just before the COVID‐19 outbreak.

A subset of children with severe asthma treated with Omalizumab, a biological agent included in the add‐on treatments for severe asthma, was separately analyzed comparing the level of asthma control between March‐April 2019 and 2020. These children are followed, in a dedicated outpatient clinic of our Unit of Pediatric Allergy and Respiratory Medicine every 2 or 4 weeks based on their current regimen.

Collected data were anonymized and recorded in a database. Results of GINA and RCAT scores were expressed as median and IQR and compared through non‐parametric tests (Wilcoxon test). We used Fisher exact test for comparing dichotomous data and Spearman's correlation coefficient (r_s_) for analyzing correlations.

All parents provided written informed consent to the use of clinical data for research purposes. The study was approved by the Ethics Committee of Padova General Hospital (protocol n. 0045208).

## 4 RESULTS

Ninty‐two asthmatic children (67 males) were identified, whose characteristics are listed in Table 1.

**Table 1:** Characteristics of patients in March 2020

Characteristic	Result
Number of patients (males)	92 (67)
Age (years)	12 ( ± 3)
Nasal swab for SARS CoV 2 (pos/total performed)	0/5 (performed in 3 patients)
Allergic	85
1 allergen	25 (27.2%)
2 allergens ≥ 3 allergens^a^	15 (16.3%)
	45 (48.9%)
Therapy prescribed	
None	15 (16.3%)
ICS	24 (26.1%)
ICS + LABA	49 (53.3%)
ICS + LABA + MK	4 (4.3%)
Practice sport regularly^b^	7
Online workout class	4 (4.3%)
Outdoor workout	3 (3.3%)

Abbreviations: ICS, inhaled corticosteroids; LABA, long‐acting β2‐agonist; MK, montelukast.

^a^ From 3 to 10 allergens.

^b^ In 2019 67 children (72.3%) used to practice a sport regularly.

### 4.1 Asthma therapy

**Figure 1:**
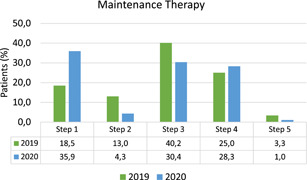
Asthma therapy taken: steps established according to GINA guidelines.^6^

The distribution of the maintenance therapy according to GINA steps^7^ in 2019 and 2020 is reported in Figure 1.

We evaluated whether recruited children followed the indications for the maintenance therapy given in the previous visit or whether they modified the therapy, spontaneously or according to family pediatrician's suggestion.

In 2019 (Table 2) 78/92 patients (84.8%) took the maintenance therapy as prescribed, while in 2020 (Table 3) only 57/92 patients (62%) took the maintenance therapy as prescribed.

**Table 2:** Therapy prescribed and taken in 2019

	Prescribed				
Taken	Step 1	Step 2	Step 3	Step 4	Step 5
Step 1	**8**	2	5	2	0
Step 2	1	**10**	1	0	0
Step 3	1	0	**34**	2	0
Step 4	0	0	0	**23**	0
Step 5	0	0	0	0	**3**

**Table 3:** Therapy prescribed and taken in 2020

	Prescribed					
Taken	Step 1	Step 2	Step 3		Step 4	Step 5
Step 1	**13**	7	8		5	0
Step 2	0	**3**	1		0	0
Step 3	2	0	**24**		2	0
Step 4	2	2	4		**16**	2
Step 5	0	0	0		0	**1**

Compared to 2019, in 2020 a higher proportion of children modified their maintenance therapy (35/92 [38%] vs. 14/92 [15.2%], *p* < .001), with a significant increase in both the proportion of those who increased and those who decreased the therapy. In fact, a reduction in maintenance therapy was reported in 25/92 children (27.2%) in 2020 and in 12/92 (13%) of children in 2019 (*p* = .026); on the other hand, an increase was reported in 10/92 children (10.9%) in 2020 and in 2/92 children (2.2%) in 2019 (*p* = .033).

### 4.2 Asthma control

**Figure 2:**
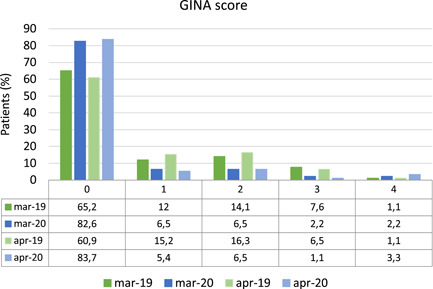
Asthmatic children distribution according to GINA score^6^

In the analysis of asthma control, the GINA score was calculated in March and April 2019 and in March and April 2020, as detailed in Figure 2. Considering March, in 2020, 53 children (57.6%) showed the same level of asthma control, 27 (29.3%) a better asthma control and 12 (13%) worse asthma control. Considering April, in 2020 53 children (57.6%) showed the same level of asthma control, 30 (32.6%) a better asthma control in 2020, and 9 (9.8%) worse asthma control. As for the whole group of children, the GINA score resulted significantly lower (indicating a better control) in March 2020 compared to March 2019 (*p* = .023) and in April 2020 compared to April 2019 (*p* = .007). A significant inverse correlation was described between GINAscore and ACT both in March 2020 (*r*_s_ = −0.613, *p* < .001) and in April 2020 (*r*_s_ = −0.629, *p* < .001).

Ten AEs were reported in March/April 2019 while four AEs were reported in March/April 2020 (*p* = .095). In 2019, six patients had each one AE and two patients had each two AEs; two patients were admitted to the Emergency Department for less than 24 h. In 2020, four patients had each one AE and none was admitted in the Emergency Department; among these four patients only one increased the therapeutic step during 2020, while the remaining three did not modified the previously prescribed therapy.

### 4.3 Interaction between asthma control and therapy

In 2020, the 25 children who reduced the maintenance therapy, compared to the remaining 67 children, showed no difference in the level of control as assessed by GINA score calculated either in March 2020 (*p* = .417) or in April 2020 (*p* = .465).

Likewise, the 10 children who increased the maintenance therapy, compared to the remaining 82 children, showed no difference in the level of control as assessed by GINA score calculated either in March 2020 (*p* = .31) or in April 2020 (*p* = .654).

In the 10 children who increased the therapy in 2020, the GINA score resulted significantly higher in March 2020 than in March 2019 (*p* = .041), while no significant difference was found between April 2020 and April 2019 (*p* = .139).

On the other hand, in the 25 children who reduced the therapy in 2020, no significant difference in GINAscore was found between 2020 and 2019 calculated both in March (*p* = .620) and in April (*p* = .103).

Finally, if we consider only the 57 children who did not modify the therapy, no significant difference was found in GINAscore between 2020 and 2019 (March *p* = .172, April *p* = .140).

### 4.4 Rhinitis control

**Figure 3:**
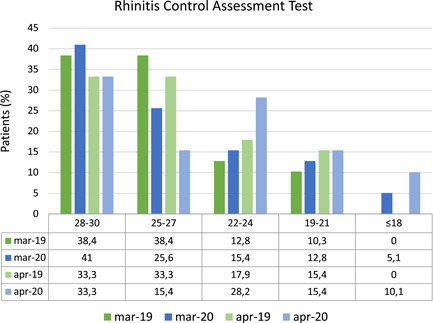
Rhinitis control

In a subgroup of 39 children older than 12 years old, who suffered also from allergic rhinitis, we evaluated rhinitis control (Figure 3). No significant difference was found comparing the RCAT score in 2020 and 2019 (March 2020: 26.13 [IQR 23.25–30] vs. March 2019: 27 [IQR 25–30], *p* = .684; April 2020: 25 [IQR 22–30] vs. April 2019: 27 [IQR 23.25–30], *p* = .290).

The correlation between GINAscore and RCATscore was not significant in all the months evaluated (*p* > .05).

Likewise, considering only the subgroup of 28 children with seasonal allergic rhinitis sensitized to *Graminaceae, Cynodon, Plantago lanceolate*, no significant correlation between GINAscore and RCATscore was found (*p* > .05).

### 4.5 Severe asthma treated with omalizumab

Thirteen children (nine males) with severe asthma treated with Omalizumab (mean age 13.7 ± 2.2) have been followed in our outpatient clinic during the analyzed period. As for sensitization to perennial allergens, 10 patients were sensitized to dust mites, 1 to mold and 2 to dog and cat fur. None of the 13 children performed COVID‐19 nasal swab, none presented with asthma exacerbations in March/April 2020, all took the maintenance therapy as prescribed (GINA step 5, high dose inhaled corticosteroids and long‐acting β2‐agonists). In March 2020, asthma symptoms were well‐controlled (GINAscore = 0) in 9 children (69.2%), partially‐controlled (GINAscore = 2) in 3 children (23.1%) and uncontrolled (GINAscore = 4) in 1 child (7.7%). In April 2020 asthma symptoms well‐controlled (GINAscore = 0) in 11 children (84.6%), partly‐controlled (GINAscore = 2) in 1 child (7.7%) and uncontrolled (GINAscore = 4) in 1 child (7.7%).

GINA score resulted significantly lower in March 2020 than in March 2019 (*p* = .011) and in April 2020 than in April 2019 (*p* = .017). A significant correlation was found between GINAscore both in March 2020 (*r*_s_ = −0.490; *p* = .045) and in April 2020 (*r*_s_ = −0.580; *p* = .019) and the number of months from the first administration of Omalizumab.

### 4.6 Psychological functioning during the lockdown

SDQ was administered to a subset of 45 children from the May 28th to July 23rd 2020, selected on the base of the age range for which the questionnaire was developed. Most of the asthmatic children reported symptoms within the normal range, thus TDS resulted normal in 97.8% of the analyzed children. Considering the different five subscales, the questionnaire showed normal ratings as follows: EMO 100%, COND 95.6%, HYPER 91.1%,_PEER 97.8%,_PROS 100%. A significant positive correlation was found between the GINA test and SDQ‐emotional symptoms (*r* = .299), while no other significant correlations were found.

## 5 DISCUSSION

This study analyzed the impact of the COVID‐19 pandemic and lockdown in asthmatic children. The lockdown had an impact on children's approach to their maintenance therapy, with heterogeneous effects: compared to the previous year, in fact, an increased proportion of children took a daily therapy higher than prescribed and an increased proportion of children took a daily therapy lower than prescribed. As far as asthma control is concerned, we found that the level of asthma control was significantly improved during the lockdown compared to the same period of the previous year.

In the 92 asthmatic children included in the study, we showed that compared to 2019, in 2020 a higher proportion of children modified their maintenance therapy spontaneously. Intriguingly the lockdown affected differently children's attitudes toward their maintenance therapy. In fact, on one hand, an increased proportion of children reduced their maintenance therapy, likely as an effect of the improved overall asthma control due to the reduced exposure to main asthma triggers during the confinement period. On the other hand, also the proportion of children who stepped up their therapy increased during the lockdown. Interestingly the level of asthma control in this subgroup of children was worse in March 2020 than in March 2019, while no differences were found comparing the months of April. This finding suggests that the therapy step‐up might have been guided by a worsening in asthma symptoms in the first months of 2020. In addition, although no specific signals emerged in the analysis of the psychological questionnaire in this subgroup of patients, we cannot exclude the fact that fear and anxiety related to COVID‐19 pandemic could have a role in guiding the increase in maintenance therapy. In fact, in the initial phases of the pandemic, patients with chronic lung diseases, including moderate‐severe asthma and allergy were considered at a potential higher risk of developing severe COVID‐19 than otherwise healthy people.^6^

In addition, also the fear of having limited access to hospital facilities may have induced some patients to increase their therapy.^13^

Comparing the lockdown period in March and April 2020 with the same 2‐month period of 2019, we found a better level of asthma control in about one‐third of children. This is in line with the findings of a recent global survey, which reports that while most children showed the usual level of asthma control during the COVID 19 pandemic, 20% showed a control better than expected with a 2.60 risk ratio of having a better than expected versus worse than expected level of control.^14^ This result is likely ascribable to the reduced exposure to main asthma trigger factors, such as viral infections, outdoor allergens, physical activities, and air pollution.^13^, ^15^, ^16^, ^17^

Furthermore, compared to March/April 2019, a lower number of AEs were reported in March/April 2020, and none of the patients was admitted to the Emergency Department. This finding may be related to a less severe presentation of symptoms but it might also be explained by caregivers’ reticence to bring children to the ER because of the risk of exposure to SARS‐CoV‐2 in a health‐care setting.^18^, ^19^

The subgroup of children who were asked for psychological symptoms did not report clinical values, falling in the normative range except for one case. The association between a poor asthma control and an increase of emotional fatigue, such as sadness or worries, deserves further investigations. First, studying the direction of this association, secondly deepening the role of the COVID‐19 pandemic in impacting on the perceived emotional stress reported by children and maybe mediating or moderating this association.

We separately analyzed the 13 children with severe asthma treated with omalizumab, followed in our outpatient clinic during the analyzed period. Also, in this group of patients, we found that symptom control was better in 2020. Again, this result could be related to the reduced exposure to typical asthma triggers, but it may also be explained by the effectiveness of the biological drug on the overall level of asthma control, as suggested by the correlation between the level of control and the number of months since the first omalizumab administration. In keeping with this result, the World Allergy Organization advised to continue administering biological therapies during the COVID‐19 pandemic in patients for whom such therapies are clearly indicated and have been effective^20^; likewise, a similar indication is reported in the recent ARIA‐EAACI statement.^21^

In the analysis of allergic rhinitis, no significant differences were found comparing symptoms control in 2020 and 2019, either considering all the children with allergic rhinitis or considering only those sensitized to *Graminaceae, Cynodon, Plantago lanceolate*. The seasonal pollen monitoring in our Region (Veneto) showed similar low levels in March 2019 and March 2020; in April the seasonal monitoring showed high levels during most of the month in 2020, while they became high only after the 13th in 2019, suggesting a potential higher exposure in 2020.^22^ In contrast, we found no difference between 2019 and 2020 in rhinitis symptoms, likely as an effect of the limited outdoor life because of the confinement.

The main limitation of our study is that the assessment of asthma control was only based on the symptoms reported by patients and their parents. In particular, we could not include spirometric data as lung function tests were stopped during the first acute phase of the pandemic. In addition, the included children belong to a selected population of asthmatic children referred to a third‐level center, so they cannot be considered representative of the average pediatric asthma patient. Nonetheless, as the level of severity is, on average, higher in our patients, we can speculate that the study if conducted in the general pediatric population would have led to similar or even more reassuring results.

In conclusion, the COVID‐19 pandemic lockdown in north‐east Italy had a significant impact on asthmatic children with respect to both the level of disease control and the approach to maintenance therapy. The level of control resulted improved during the lockdown period, likely because of the reduced exposure to typical asthma triggers due to confinement. As for the maintenance treatment, some patients reduced it, likely because of the good level of disease control, while others increased it, either because of symptoms or because of fear and anxiety related to the spreading of the COVID‐19 pandemic.

## CONFLICT OF INTERESTS

The authors declare that there are no conflict of interests.
